# 固相萃取-气相色谱-静电场轨道阱高分辨质谱测定海水中4类34种含氯持久性有机污染物

**DOI:** 10.3724/SP.J.1123.2024.07017

**Published:** 2025-04-08

**Authors:** Menghao GAO, Xiaoying LI, Yuan GAO, Haijun ZHANG, Jiping CHEN

**Affiliations:** 1.大连海事大学环境科学与工程学院,辽宁 大连 116026; 1. College of Environmental Sciences and Engineering, Dalian Maritime University, Dalian 116026, China; 2.中国科学院大连化学物理研究所,辽宁 大连 116023; 2. Dalian Institute of Chemical Physics, Chinese Academy of Sciences, Dalian 116023, China

**Keywords:** 固相萃取, 气相色谱-静电场轨道阱高分辨质谱, 含氯持久性有机污染物, 海水, solid-phase extraction (SPE), gas chromatography-electrostatic field orbitrap high resolution mass spectrometry (GC-Orbitrap-HRMS), chlorinated persistent organic pollutants (POPs), seawater

## Abstract

海洋在新污染物的环境迁移、转化过程中起到重要作用。海水中新污染物的准确定量是厘清其环境行为、评估其环境风险的基础。本研究采用液液萃取和固相萃取建立海水样品前处理方法,基于气相色谱-静电场轨道阱高分辨质谱(GC-Orbitrap-HRMS)测定海水中4类34种含氯持久性有机污染物(POPs)的定量分析方法,即:25种有机氯农药(OCPs)、6种多氯联苯(PCBs)同系物、短链氯化石蜡(SCCPs)和2种德克隆(DPs)同分异构体。通过优化液液萃取提取溶剂种类,选择二氯甲烷进行萃取。随之对固相萃取不同洗脱溶剂进行评价,最终使用体积比为9∶1的正己烷和丙酮混合溶剂进行洗脱。质谱采用电子轰击源(EI)(正离子模式)监测OCPs和PCBs目标化合物离子,采用负化学源(NCI)监测SCCPs和DPs,内标法定量。结果表明,所建立的方法具有较低的检出限,34种含氯POPs检出限为0.006~2.78 ng/L,定量限为0.02~11.12 ng/L;方法准确度和精密度通过测定目标化合物的加标回收率得到验证,低、中、高3个加标水平下的回收率为70.6%~128.9%,相对标准偏差(*n*=6)为0.2%~19.2%。通过实际海水样品分析显示,SCCPs的检出率和浓度水平最高,质量浓度最高为130.6 ng/L,需要持续重点关注。该方法前处理操作简便,灵敏度高,样品需求量少,适用于大批量海水样本中多种含氯POPs的痕量检测。

## Abstract (en)

Ocean acts as a “sink” for pollutants in the natural environment. Consequently, issues focused on marine pollution from terrestrial origin is attracting increasing attention. Persistent organic pollutants (POPs), including organochlorine pesticides (OCPs), polychlorinated biphenyls (PCBs), short-chain chlorinated paraffins (SCCPs), and dechlorane plus isomers (DPs), are serious hazards for both the environment and humans. These POPs have been widely detected in the marine environment and are typically present at trace levels; however, separating and determining individual contaminants require large amounts of sampling and time. Establishing an accurate analytical method for determining typical POPs is critical for studying their environmental behavior and associated ecological risks to the marine environment.

In this study, we developed a method based on solid-phase extraction (SPE) combined with gas chromatography-electrostatic field orbitrap high resolution mass spectrometry (GC-Orbitrap-HRMS) for determining 34 chlorinated POPs in seawater, including 25 OCPs, six PCB congeners, SCCPs and two DPs. The chromatographic conditions and MS parameters were optimized, and the effects of the extraction solvent and purification method were systematically studied. Dichloromethane exhibited satisfactory extraction efficiencies during the liquid-liquid extraction (LLE) of seawater samples, with recoveries of 73.1%-120.5% for OCPs, 87.2%-101.7% for PCBs, 105.5% for SCCPs, and 74.9%-78.6% for DPs, respectively. Purification using a SPE column with 500 mg of Florisil was adopted, and 9∶1 (v/v) *n*-hexane/acetone was confirmed as the eluent with recoveries between 68.2% and 122.8% for all the 34 chlorinated POPs. A DB-5MS (15 m×0.25 mm×0.10 μm) capillary chromatographic column was used to separate the target compounds, with an electron ionization (EI) source used to detect OCPs and PCBs, whereas SCCPs and DPs were determined in negative chemical ionization (NCI) source. All target compounds were analyzed in full-scan mode. An internal standard quantification method was used for OCPs and SCCPs while isotope dilution quantification was used for PCBs and DPs. The severe interference observed during the detection of chlorinated POPs in the mixture of co-extracted substances was completely eliminated following the purification.

The 34 target chlorinated POPs exhibited good linearities in their corresponding ranges, with correlation coefficients (*R*^2^) exceeding 0.9. The method demonstrated low detection limits under the optimized conditions, with values of 0.009-0.061 ng/L for the 25 OCPs, 0.006-0.016 ng/L for the six PCBs, 2.78 ng/L for the SCCPs, and 0.021-0.023 ng/L for the two DPs, with lower limits of determination of 0.06-0.24, 0.02-0.06, 11.12, and 0.08-0.09 ng/L, respectively. Accuracy and precision were validated by the recoveries of samples spiked at low, medium, and high levels, which ranged between 70.6% and 128.9%. Relative standard deviations (*n*=6) were determined to be 0.2%-19.2%. These results highlight the suitability of the developed method for analyzing trace amounts of chlorinated POPs in seawater. The method is characterized by simple sample pretreatment, high sensitivity, fast analytical throughput, cost-effectiveness, and good stability for trace-level detection; hence, it is suitable for the rapid and accurate analysis of typical chlorinated POPs in seawater. This method is expected to play a significant role in marine environmental monitoring and the emergency surveillance of seawater pollution. The developed method was applied to seawater samples collected from Bohai, which revealed that the highest detection frequency (90%) was recorded for the SCCPs, while *α*-hexachlorocyclohexane (*α*-HCH) was only detected in 30% of the samples. All other OCPs were below the detection limit. PCB-52 was the only PCB congener detected in the seawater samples. The SCCPs were detected in much higher concentrations than the other POPs, with the highest value of 130.6 ng/L recorded. Consequently, particular attention must be paid to SCCPs.

随着全球传统持久性有机污染物(persistent organic pollutants, POPs)的限制和/或自愿退出或淘汰,一些含氯新污染物对公众健康和生态环境的危害正逐步显现,且多以痕量或超痕量水平存在于环境介质中,具有环境持久性和生物毒性,其引起的生态风险和人体健康风险具有隐蔽性^[1⇓⇓-4]^。海洋作为自然环境中污染物的“汇”^[5]^,传统管控POPs化合物如有机氯农药(organochlorine pesticides, OCPs)、多氯联苯(polychlorinated biphenyls, PCBs)和新污染物如短链氯化石蜡(short-chain chlorinated paraffins, SCCPs)与德克隆(dechlorane plus, DPs)等沿陆海多介质迁移,进而对海洋生态环境带来巨大的安全风险和生态健康威胁。其中,SCCPs和DPs分别在2017年和2023年被列入斯德哥尔摩公约附件A进行管控^[6,7]^。分析检测含氯POPs、研究其环境迁移转化行为、识别其毒性并进行风险评估,是加强海洋生态环境中含氯POPs化合物管理和制定有效污染防治措施的关键基础。

当前国内外对OCPs、PCBs、SCCPs和DPs的分析检测中,广泛针对单一种类目标化合物采用色谱或色谱-质谱联用技术进行分析,如气相色谱-串联质谱法(GC-MS/MS)^[8]^、气相色谱法(GC)^[9]^、气相色谱-质谱法(GC-MS)^[10,11]^、液相色谱-高分辨质谱法(LC-HRMS)^[12]^和液相色谱-串联质谱法(LC-MS/MS)^[13]^等。传统分析方法通常用于单一种类化合物检测,多采用液液萃取/固相萃取提取(样品量至少1 L),硅胶、氧化铝玻璃层析柱或联用方式净化处理^[14⇓⇓⇓⇓⇓⇓-21]^,这些方法净化效果好,但分析流程复杂,耗时长,分析检测难以满足大批量样品的快速检测需求。因此,开发一种能够准确区分并定量4类含氯POPs的分析方法,对于提高实验室效率、降低时间和人力成本具有重要意义。由于SCCPs的化学多样性(包含成千上万个同分异构体),传统的质谱技术在分辨率上可能不足以实现对这些复杂混合物中各个组分的精确分析。因此,采用高分辨率质谱技术,如气相色谱-静电场轨道阱高分辨质谱(GC-Orbitrap-HRMS)对于提高这些含氯化合物分析的精度至关重要,这不仅确保了结果的可靠性和准确性,还显著提高了分析效率。GC-Orbitrap-HRMS以其高灵敏度和分辨率,在快速分析多种污染物方面展现出显著优势,已被广泛应用于各类复杂基质中污染物的定性与定量分析^[22⇓⇓-25]^。在处理复杂的海水基质时,GC-Orbitrap-HRMS也能够提供精确的定性和定量结果,能有效识别和测量海水中的污染物。

本研究拟建立一种液液萃取-固相萃取净化的样品前处理方法,对海水中25种OCPs、6种PCBs同系物、SCCPs和2种DPs同分异构体这4类含氯POPs进行统一提取,采用GC-Orbitrap-HRMS分别在电子轰击源(electron ionization, EI)和负化学源(negative chemical ionization, NCI)条件下进样分析,实现对海水中4类34种含氯POPs的准确定量分析。

## 1 实验部分

### 1.1 仪器、试剂与材料

Q Exactive GC-Orbitrap高分辨质谱仪(配备AI1310自动进样器和TRACE 1310气相色谱仪,美国Thermo Fisher公司);色谱柱DB-5MS(15 m×0.25 mm×0.10 μm,固定相为苯基芳基聚合物,美国Agilent公司);R-300旋转蒸发仪(瑞士BuChi公司), GTCS-2013B往复振荡仪(北京国环高科自动化技术研究院),DC-12氮吹浓缩仪(上海安普公司); Milli-Q超纯水机(德国Merck Millipore公司)。

Florisil固相萃取柱(6 mL/500 mg,上海安普公司);丙酮(成都科隆公司)、正己烷和二氯甲烷(美国Honeywell公司)均为农残级;无水硫酸钠(分析纯,天津大茂公司)。实验用水为Milli-Q超纯水。

标准品:有机氯农药混合标准品购自坛墨质检(常州);多氯联苯P48-M-CS1、P48-M-CS2、P48-M-CS3、P48-M-CS4和德克隆*syn*-DP、*anti*-DP购自Wellington Laboratories公司(Ontario,加拿大); SCCPs标准溶液(氯含量分别为51.5%、55.5%、63%, 100 ng/μL环己烷)购自Dr. Ehrenstorfer公司(Augsburg,德国)。替代物/同位素内标:^13^C_6_-*δ*-六氯环己烷购自Cambridge Isotope Laboratories(Andover,美国);四氯间二甲苯和十氯联苯混合标准溶液购自Dr. Ehrenstorfer, P48-M-ES和^13^C_10_-*anti*-DP购自Wellington Laboratories。回收率内标:^13^C_6_-六氯苯和P48-RS分别购自CIL和Wellington Laboratories;氘代1,4-二氯苯、氘代菲和氘代$\overset{艹}{屈}$ 混合溶液购自Dr. Ehrenstorfer。配制工作液时,取一定量的标准储备液,用正己烷或壬烷定容后使用,于4 ℃冰箱冷藏保存。

替代物/同位素内标混合溶液配制:取一定量的替代物/同位素内标,加入正己烷稀释,得到质量浓度分别为500 μg/L的^13^C_6_-*δ*-六氯环己烷、50 μg/L的四氯间二甲苯和十氯联苯、100 μg/L的P48-M-ES和500 μg/L的^13^C_10_-*anti*-DP的混合溶液。回收率内标混合溶液配制:取一定量的回收率内标,加入壬烷稀释,得到质量浓度分别为10 μg/L的^13^C_6_-六氯苯、200 μg/L的氘代1,4-二氯苯、氘代菲和氘代$\overset{艹}{屈}$ 和50 μg/L的P48-RS的混合溶液。

### 1.2 样品采集

在2023年7月采集渤海近岸海水样品,采集完毕后将海水保存在2 L的棕色样品瓶中,于4 ℃冰箱中冷藏保存,14天内完成样品前处理。

### 1.3 样品前处理

海水样品经0.45 μm玻璃纤维膜过滤,然后准确量取1 L样品于分液漏斗,加入20 μL替代物/同位素内标混合溶液,振荡混匀,加入50 mL二氯甲烷,摇匀,待其无气体放出后置于往复振荡仪上振荡15 min,用玻璃烧瓶接取下层有机相,重复上述步骤3次,收集所有萃取液,加入无水硫酸钠脱水,旋转蒸发浓缩至0.5~1 mL,加入20 mL正己烷置换溶剂,再次浓缩至1 mL。参考HJ 699-2014方法进行样品净化:上样前先用8 mL正己烷活化Florisil固相萃取柱,将浓缩液转移至柱头,用3 mL正己烷洗涤玻璃烧瓶3次,洗涤液一并转移至柱头,用10 mL正己烷-丙酮混合溶剂(9∶1, v/v)洗脱,收集洗脱液,氮气吹扫浓缩至近干,加入回收率内标混合溶液定容至20 μL,涡旋混匀后待上机分析。

### 1.4 质量控制和保证

实验过程中避免接触塑料制品,所有玻璃器皿使用前均使用二氯甲烷润洗3次。无水硫酸钠使用前在650 ℃下灼烧5 h并置于干燥器保存。空白实验采用超纯水依照以上处理步骤进行样品前处理及仪器分析,每10个海水样品编入一个空白样品(超纯水),以监测实验过程中可能产生的污染。

### 1.5 仪器分析

色谱条件 DB-5MS毛细管柱(15 m×0.25 mm×0.10 μm),进样口温度280 ℃,采用分流进样方式,分流比5∶1,进样量1 μL,载气为氦气(纯度>99.9%),载气流速1 mL/min。对于OCPs和PCBs,程序升温条件如下:110 ℃保持1 min,以40 ℃/min升至200 ℃,以10 ℃/min升至330 ℃,保持5 min。对于SCCPs和DPs,程序升温条件如下:100 ℃保持2 min,以20 ℃/min升至160 ℃,保持2 min, 30 ℃/min升至310 ℃,保持10 min。

质谱条件 采用电子轰击源(EI)检测OCPs和PCBs,离子源温度250 ℃,电子能量70 eV,质量扫描区间为*m/z* 100~1000;采用负化学电离源检测SCCPs和DPs,离子源温度为220 ℃,以甲烷(纯度>99.9%)作为反应气,流速2 mL/min,质量扫描区间为*m/z* 200~1000。传输线温度为280 ℃。定量、定性离子等参数见表1。

**表1:** 有机氯农药、多氯联苯、短链氯化石蜡及德克隆的定量、定性离子及GC保留时间

Classification	No.	Compound	Abbreviation	Formula	Quantitative ion (*m/z*)	Qualitative ion (*m/z*)	Retention time/min	Surrogate/ isotope
OCPs	1	*α*-hexachlorocyclohexane	*α*-HCH	C_6_H_6_Cl_6_	180.93727	182.93431	7.20	tetrachloro-
	2	*γ*-hexachlorocyclohexane	*γ*-HCH	C_6_H_6_Cl_6_	180.93721	218.91110	7.44	*m*-xylene/
	3	*β*-hexachlorocyclohexane	*β*-HCH	C_6_H_6_Cl_6_	180.93698	218.91103	7.53	decachlorobi
	4	*δ*-hexachlorocyclohexane	*δ*-HCH	C_6_H_6_Cl_6_	180.93724	218.91103	7.73	-phenyls
	5	pentachloronitro-benzene	PCNB	C_6_Cl_5_NO_2_	236.84073	234.84354	7.60	
	6	heptachlor	HEP	C_10_H_5_Cl_7_	271.80969	273.80664	8.27	
	7	aldrin	ALD	C_12_H_8_Cl_6_	262.85651	264.85355	8.60	
	8	heptachlor epoxide	HeEp	C_10_H_5_Cl_7_O	352.84366	354.84045	8.97	
	9	*trans*-heptachlorepoxide	*trans*-HeEp	C_10_H_5_Cl_7_O	352.84378	252.99953	9.01	
	10	*trans*-chlordane	*trans*-CD	C_10_H_6_Cl_8_	372.82574	374.82242	9.19	
	11	*cis*-chlordane	*cis*-CD	C_10_H_6_Cl_8_	372.82556	374.82251	9.35	
	12	methoxychlor	MeOCl	C_16_H_15_Cl_3_O_2_	227.10649	228.10957	11.18	
	13	*p*,*p'*-dichlorodiphenyldichloroethylene	*o*,*p*'-DDE	C_14_H_8_Cl_4_	245.99948	247.99658	9.27	
	14	*p*,*p'*-dichlorodiphenyldichloroethylene	*p*,*p*'-DDE	C_14_H_8_Cl_4_	245.99973	247.99677	9.59	
	15	*o*,*p*'-dichlorodiphenyldichloroethane	*o*,*p*'-DDD	C_14_H_10_Cl_4_	235.00746	237.00455	9.66	
	16	*p*,*p*'-dichlorodiphenyldichloroethane	*p*,*p*'-DDD	C_14_H_10_Cl_4_	235.00754	237.00455	10.01	
	17	*o*,*p'*-dichlorodiphenyltrichloroethane	*o*,*p*'-DDT	C_14_H_9_Cl_5_	235.00748	237.00455	10.06	
	18	*p*,*p'*-dichlorodiphenyltrichloroethane	*p*,*p*'-DDT	C_14_H_9_Cl_5_	235.00745	237.00461	10.45	
	19	dieldrin	DIEL	C_12_H_8_Cl_6_O	262.85645	379.86703	9.57	
	20	endrin	END	C_12_H_8_Cl_6_O	262.85669	244.95012	9.78	
	21	endrin aldehyde	EndAl	C_12_H_8_Cl_6_O	344.89813	346.89517	10.06	
	22	endrin ketone	EndKet	C_12_H_9_Cl_5_O	316.90356	314.90643	10.86	
	23	endosulfan sulfate	EndoSu	C_9_H_6_Cl_6_O_4_S	271.80954	273.80679	10.33	
	24	*β*-endosulfan	*β*-Endo	C_9_H_6_Cl_6_O_3_S	236.84096	338.87268	9.86	
	25	*α*-endosulfan	*α*-Endo	C_9_H_6_Cl_6_O_3_S	236.84091	238.83800	9.31	
PCBs	26	2,4,4'-trichlorobiphenyl	PCB 28	C_12_H_7_Cl_3_	255.96085	257.95792	8.14	^13^C_12_-PCB 28
	27	2,2',5,5'-tetrachlorobiphenyl	PCB 52	C_12_H_6_Cl_4_	291.91864	289.82139	8.48	^13^C_12_-PCB 52
	28	2,2',4,5,5'-pentachlorobiphenyl	PCB 101	C_12_H_5_Cl_5_	325.87991	327.87704	9.31	^13^C_12_-PCB 101
	29	2,2',3,4,4,5'-hexachlorobiphenyl	PCB 138	C_12_H_4_Cl_6_	359.84097	361.83801	10.21	^13^C_12_-PCB 138
	30	2,2',4,4',5,5'-hexachlorobiphenyl	PCB 153	C_12_H_4_Cl_6_	359.84100	361.82807	10.51	^13^C_12_-PCB 153
	31	2,2',3,4,4',5,5'-heptachlorobiphenyl	PCB 180	C_12_H_3_Cl_7_	393.80176	395.79919	11.37	^13^C_12_-PCB 180
SCCPs	32	short-chain chlorinated paraffins	SCCPs	C_10_H_17_Cl_5_	279.00604	277.00899	6.0-7.5	^13^C_6_-*δ*-HCH
				C_10_H_16_Cl_6_	312.96706	314.96411	6.5-9.0	
				C_10_H_15_Cl_7_	346.92809	348.92514	7.5-9.5	
				C_10_H_14_Cl_8_	380.88912	382.88617	8.5-10.0	
				C_10_H_13_Cl_9_	416.84720	414.85015	9.0-10.0	
				C_10_H_12_Cl_10_	450.80823	448.81118	9.5-10.5	
				C_11_H_19_Cl_5_	293.02169	291.02464	7.0-8.5	
				C_11_H_18_Cl_6_	326.98271	328.97976	7.0-8.5	
				C_11_H_17_Cl_7_	360.94374	362.94079	7.5-9.5	
				C_11_H_16_Cl_8_	394.90477	396.90182	8.5-10.0	
				C_11_H_15_Cl_9_	430.86285	428.86580	9.0-10.5	
				C_11_H_14_Cl_10_	464.82388	462.82683	10.0-11.0	
				C_12_H_21_Cl_5_	307.03734	305.04029	7.5-9.0	
				C_12_H_20_Cl_6_	340.99836	342.99541	8.0-10.0	
				C_12_H_19_Cl_7_	374.95939	376.95644	9.0-10.5	
				C_12_H_18_Cl_8_	408.92042	410.91747	9.5-11.0	
				C_12_H_17_Cl_9_	444.87850	442.88145	9.5-11.0	
				C_12_H_16_Cl_10_	478.83953	476.84248	10.0-11.0	
				C_13_H_23_Cl_5_	321.05299	319.05594	8.0-9.5	
				C_13_H_22_Cl_6_	355.01401	357.01106	8.5-10.5	
				C_13_H_21_Cl_7_	388.97504	390.97209	9.0-11.0	
				C_13_H_20_Cl_8_	422.93607	424.93312	9.5-11.0	
				C_13_H_19_Cl_9_	458.89415	456.89710	10.0-11.5	
				C_13_H_18_Cl_10_	492.85518	490.85813	10.0-11.5	
DPs	33	*syn*-dechlorane plus	*syn*-DP	C_18_H_12_Cl_12_	653.71136	651.71466	12.17	^13^C_10_-*anti*-DP
	34	*anti*-dechlorane plus	*anti*-DP	C_18_H_12_Cl_12_	653.71271	651.71454	12.31	

## 2 结果与讨论

### 2.1 质谱条件优化

EI源作为最常用的电离源,广泛应用于环境中OCPs、PCBs等POPs的检测。但当使用EI源分析SCCPs时,EI源轰击能量过高使得SCCPs在电离过程中发生多种碎裂反应,产生大量*m/z*较小的离子碎片,质谱图复杂且缺乏特征性碎片离子^[26]^。当选择甲烷作为反应气的NCI源,SCCPs主要产生[M-Cl]^-^的特征离子峰。相似地,DPs通常也使用NCI源进行定量^[27]^,因此本实验采用NCI源监测SCCPs和DPs。

采用DB-5MS毛细管色谱柱对目标物进行分离,MS全扫描检测,选取相对丰度最高的离子碎片作为定量离子,相对丰度次高的离子碎片作为定性离子(表1)。EI条件下25种OCPs和6种PCBs的提取特征离子色谱图见图1a,NCI条件下SCCPs和2种DPs同分异构体的[M-Cl]^-^提取特征离子色谱图见图1b。

**图1:**
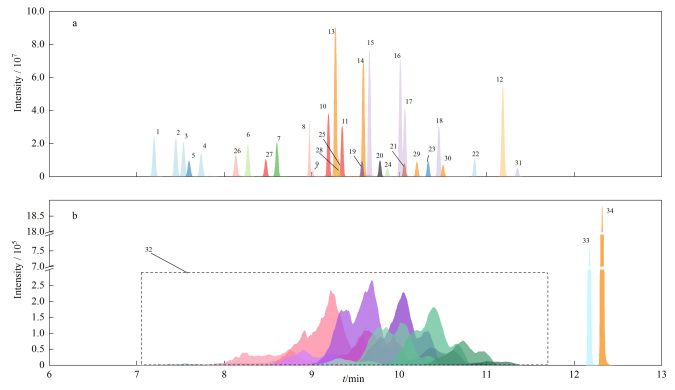
(a)25种有机氯农药和6种多氯联苯在EI条件下和(b)短链氯化石蜡和2种德克隆同分异构体在NCI条件下的提取离子色谱图

### 2.2 萃取溶剂的选择

基于文献[28,29]及环境标准(HJ 699-2014),本实验采用正己烷和二氯甲烷作为提取溶剂用于水样中含氯POPs的液液萃取。本实验分别考察了正己烷和二氯甲烷的萃取效率,因为海水含盐率较高,正己烷在萃取后分层耗时较长,且易出现蛛网现象,故最终选取二氯甲烷作为萃取剂,并考察了4类含氯POPs的萃取回收率。结果表明,采用二氯甲烷作为液液萃取的提取溶剂,OCPs、PCBs、SCCPs和DPs的平均回收率(*n*=3)分别为73.1%~120.5%、87.2%~101.7%、105.5%和74.9%~78.6%,萃取效果整体较优;4种POPs的提取回收率均在70%~130%范围内,满足行业标准对新污染物提取回收率的要求。

### 2.3 洗脱溶剂的选择

目标组分包含多种化合物,且化合物理化性质差异较大,为获得较高的净化效率和目标化合物的回收率,本研究共考察了3种不同比例的溶剂的洗脱效果,分别为体积比为1∶1的正己烷-二氯甲烷(A)、体积比为9∶1的正己烷-丙酮(B)、体积比为1∶1的正己烷-丙酮(C),溶剂体积均为10 mL。OCPs、PCBs、SCCPs和DPs的回收率如图2所示,当洗脱溶剂为A和C时,4组化合物的回收率较高,但部分极性化合物也会随之被洗脱下来,导致部分化合物受到干扰,回收率偏高,如异狄氏醛(EndAl),回收率分别达到176.8%和166.5%,影响分析结果的准确性。当洗脱溶剂为B时,OCPs、PCBs、SCCPs和DPs的回收率分别为68.2%~122.8%、99.4%~107.3%、105.0%和84.9%~91.6%,所有化合物的回收率整体处于可接受范围,故本实验选择溶剂B为Florisil固相萃取柱的洗脱溶剂。

**图2:**
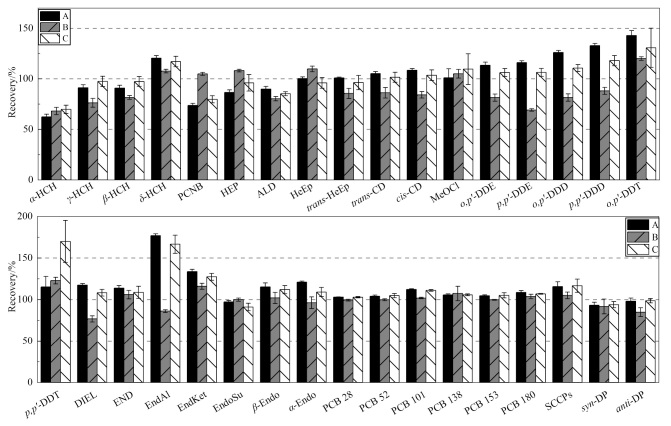
不同洗脱溶剂对有机氯农药、多氯联苯、短链氯化石蜡和德克隆回收率的影响(*n*=3)

### 2.4 线性范围和检出限

采用内标法对海水中的目标化合物进行定量,6种PCBs在1~1000 μg/L范围内线性关系良好,相关系数(*R*^2^)大于0.995; DPs在50~1000 μg/L范围内线性关系良好,*R*^2^大于0.999; 25种OCPs在20~1000 μg/L范围内呈良好的线性关系,*R*^2^均大于0.99。对于SCCPs,采用氯含量校正总响应因子定量方法制作标准曲线,以计算的氯含量(%)为横坐标,24种SCCPs同系物的总响应因子为纵坐标制作标准曲线;其中,总响应因子采用24种SCCPs同系物的相对峰面积之和与SCCPs质量浓度的比值计算获得^[30]^, *R*^2^>0.9,满足SCCPs的定量分析要求。具体的线性范围和线性回归方程等见表2。

**表2:** 有机氯农药、多氯联苯、短链氯化石蜡和德克隆的线性范围、回归方程、方法检出限和测定下限

Compound	Linear range/(μg/L)	Regression equation	*R*^2^	MDL/(ng/L)	Lower limit of determination/(ng/L)
*α*-HCH	20-1000	*y*=0.227*x*+0.014	1.000	0.017	0.07
*γ*-HCH	20-1000	*y*=0.116*x*+0.023	0.996	0.009	0.04
*β*-HCH	20-1000	*y*=0.200*x*+0.012	0.999	0.027	0.11
*δ*-HCH	20-1000	*y*=0.104*x*+0.007	1.000	0.013	0.05
PCNB	20-1000	*y*=0.108*x*-0.003	0.996	0.028	0.11
HEP	20-1000	*y*=0.203*x*-0.003	0.999	0.013	0.05
ALD	20-1000	*y*=0.198*x*+0.010	0.999	0.014	0.05
HeEp	20-1000	*y*=0.2107*x*+0.0121	0.999	0.032	0.13
*trans*-HeEp	20-1000	*y*=0.0281*x*+0.0014	0.998	0.045	0.18
*trans*-CD	20-1000	*y*=0.3336*x*+0.0184	0.999	0.033	0.13
*cis*-CD	20-1000	*y*=0.2701*x*+0.0129	0.998	0.052	0.21
MeOCl	20-1000	*y*=0.5679*x*-0.0546	0.996	0.044	0.17
*o*,*p*'-DDE	20-1000	*y*=0.8093*x*+0.0381	0.998	0.038	0.15
*p*,*p*'-DDE	20-1000	*y*=0.640*x*+0.0251	0.997	0.035	0.14
*o*,*p*'-DDD	20-1000	*y*=0.648*x*+0.0562	0.999	0.024	0.09
*p*,*p*'-DDD	20-1000	*y*=0.6097*x*+0.0437	1.000	0.027	0.11
*o*,*p*'-DDT	20-1000	*y*=0.4173*x*-0.0304	0.995	0.046	0.19
*p*,*p*'-DDT	20-1000	*y*=0.3291*x*-0.0410	0.993	0.021	0.08
DIEL	20-1000	*y*=0.0829*x*+0.0045	0.999	0.041	0.16
END	20-1000	*y*=0.0853*x*+0.0026	0.999	0.057	0.23
EndAl	20-1000	*y*=0.0381*x*+0.0055	0.998	0.048	0.19
EndKet	20-1000	*y*=0.0887*x*+0.0049	0.999	0.043	0.17
EndoSu	20-1000	*y*=0.0603*x*+0.0042	1.000	0.018	0.07
*β*-Endo	20-1000	*y*=0.0415*x*+0.0028	1.000	0.051	0.2
*α*-Endo	20-1000	*y*=0.0545*x*+0.0019	0.999	0.061	0.24
PCB 28	1-1000	*y*=0.9365*x*+0.0226	0.995	0.006	0.02
PCB 52	1-1000	*y*=0.9223*x*+0.0219	0.996	0.011	0.04
PCB 101	1-1000	*y*=0.9286*x*+0.0342	0.996	0.008	0.03
PCB 138	1-1000	*y*=1.219*x*+0.0226	0.997	0.009	0.04
PCB 153	1-1000	*y*=0.9389*x*+0.0129	0.998	0.016	0.06
PCB 180	1-1000	*y*=0.8902*x*+0.0427	0.995	0.011	0.04
SCCPs	51.5%Cl-63.0%Cl^*^	*Y*=4.71739×10^-15^×	0.938	2.78	11.12
		EXP(55.12584*X*)			
*syn*-DP	50-1000	*y*=2.4407*x*+0.0321	0.999	0.023	0.09
*anti*-DP	50-1000	*y*=1.0581*x*+0.0052	1.000	0.021	0.08

*y*: peak area of the quantitative ion of the analyte; *x*: mass concentration, ng/L. *Y*: total response factors of the SCCP mixtures; *X*: chlorine content of the SCCP mixtures. * The linear range of SCCPs was the chlorine content range of the standard solutions^[31]^.

以信噪比(*S/N*)=3计算仪器检出限,向空白样品中添加标准溶液,添加浓度为仪器检出限的3~5倍,按照样品分析的全部步骤重复7次,将各测定结果换算为样品中的浓度,计算7次平行的标准偏差(*S*)。方法检出限(method detection limit, MDL)按公式MDL=3.143×*S*计算,MDL的4倍作为测定下限(lower limit of determination)。本研究中25种OCPs、6种PCBs、SCCPs和2种DPs的MDL分别为0.009~0.061、0.006~0.016、2.78和0.021~0.023 ng/L,测定下限分别为0.06~0.24、0.02~0.06、11.12和0.08~0.09 ng/L。具体数值见表2。

### 2.5 方法的准确度和精确度

本实验采用海水基质加标来验证方法的准确度和精确度,添加低浓度(L)、中浓度(M)和高浓度(H)水平的OCPs、PCBs、SCCPs和DPs以及替代物/同位素内标混合标准溶液(目标物加标量见表3),每个加标水平平行测定6次,计算目标物的回收率以及相对标准偏差(RSD),结果见表3。低、中、高加标水平下OCPs、PCBs、SCCPs和DPs的平均回收率分别为72.3%~128.5%、72.1%~123.6%和70.6%~128.9%, RSD分别为1.1%~17.1%、0.2%~13.5%和2.7%~19.2%。替代物/同位素内标的平均回收率为70.9%~98.5%,表明该方法具有较好的重复性和稳定性,满足含氯POPs准确定量的分析要求。

**表3:** 有机氯农药、多氯联苯、短链氯化石蜡和德克隆的加标回收率及相对标准偏差(*n*=6)

Compound	Spiked/(ng/L)		Recovery/%	RSD/%	Compound	Spiked/(ng/L)		Recovery/%	RSD/%
*α*-HCH	L	0.4	78.7	3.8	*o*,*p*'-DDT	L	0.4	97.1	8.0
	M	0.5	72.7	5.8		M	0.5	103.5	4.7
	H	1	72.2	10.1		H	1	107.1	5.7
*β*-HCH	L	0.4	86.9	11.8	*p*,*p*'-DDD	L	0.4	76.2	4.7
	M	0.5	77.9	4.5		M	0.5	76.6	7.3
	H	1	79.7	5.0		H	1	77.2	4.9
*γ*-HCH	L	0.4	86.7	9.0	*p*,*p*'-DDT	L	0.4	127.7	7.9
	M	0.5	75.7	4.4		M	0.5	109.8	7.1
	H	1	76.3	3.7		H	1	108.8	5.8
*δ*-HCH	L	0.4	111.4	8.1	*p*,*p*'-DDE	L	0.4	95.1	7.0
	M	0.5	104.6	13.5		M	0.5	72.5	2.7
	H	1	113.6	6.2		H	1	76.8	5.7
PCNB	L	0.4	113.0	8.8	*trans*-CD	L	0.4	81.0	7.2
	M	0.5	103.8	6.5		M	0.5	72.6	8.2
	H	1	108.4	8.3		H	1	72.6	3.9
HEP	L	0.4	116.0	8.1	*β*-Endo	L	0.4	72.3	15.7
	M	0.5	91.0	3.5		M	0.5	82.9	8.1
	H	1	82.2	4.0		H	1	82.2	6.2
ALD	L	0.4	98.3	2.7	HeEp	L	0.4	82.0	6.1
	M	0.5	72.1	4.1		M	0.5	79.4	7.3
	H	1	73.2	3.1		H	1	75.0	6.5
*cis*-CD	L	0.4	84.6	7.0	*trans*-HeEp	L	0.4	80.2	10.9
	M	0.5	74.9	7.3		M	0.5	78.3	5.2
	H	1	75.6	5.0		H	1	85.0	15.7
DIEL	L	0.4	99.8	9.5	PCB 28	L	0.1	104.7	9.8
	M	0.5	72.8	7.4		M	0.5	107.1	0.2
	H	1	72.4	5.5		H	1	103.3	3.8
*α*-Endo	L	0.4	89.4	17.1	PCB 52	L	0.1	84.5	10.4
	M	0.5	75.2	9.6		M	0.5	87.9	4.1
	H	1	70.6	7.1		H	1	97.0	6.6
EndoSu	L	0.4	128.5	9.4	PCB 101	L	0.1	100.7	1.2
	M	0.5	121.5	6.0		M	0.5	96.5	3.4
	H	1	118.4	8.2		H	1	106.5	4.2
Endrin	L	0.4	97.0	7.7	PCB 138	L	0.1	100.8	4.2
	M	0.5	93.9	6.2		M	0.5	101.7	2.5
	H	1	83.5	5.8		H	1	78.0	4.4
EndAl	L	0.4	77.3	15.8	PCB 153	L	0.1	98.3	5.9
	M	0.5	81.1	6.9		M	0.5	104.1	0.5
	H	1	90.3	3.2		H	1	103.4	4.0
EndKet	L	0.4	72.5	10.2	PCB 180	L	0.1	93.0	4.1
	M	0.5	94.5	7.8		M	0.5	100.0	5.6
	H	1	94.4	6.7		H	1	102.6	3.2
MeOCl	L	0.4	103.1	6.0	*anti*-DP	L	0.5	74.9	7.2
	M	0.5	123.6	5.9		M	2.5	85.5	5.3
	H	1	128.9	2.7		H	5	79.3	6.5
*o*,*p*'-DDD	L	0.4	81.1	4.5	*syn*-DP	L	0.5	78.6	7.8
	M	0.5	78.5	9.0		M	2.5	91.4	7.9
	H	1	74.5	19.2		H	5	88.0	7.7
*o*,*p*'-DDE	L	0.4	94.0	1.1	SCCPs	L	50	105.5	16.5
	M	0.5	76.1	5.9	(55.5%Cl)	M	250	114.0	9.6
	H	1	70.8	5.2		H	500	80.8	5.2

L: low level; M: medium level; H: high level.

### 2.6 实际样品的分析

采用本实验方法测定了20个海水样品中的OCPs、PCBs、SCCPs和DPs。结果表明,OCPs中仅有*α*-HCH检出,质量浓度为ND(未检出)~0.3 ng/L,检出率30%,其余OCPs均低于测定下限。PCBs中仅有PCB-52检出,质量浓度为ND~0.7 ng/L。我国DP生产量及使用量较少^[32]^,在海水中仅有少量*anti*-DP检出,检出率为10%,最高质量浓度为0.3 ng/L; SCCPs的检出率最高,达到90%,质量浓度为ND~130.6 ng/L(平均49.6 ng/L),见表4。该结果低于东海海水中SCCPs的质量浓度(12.2~430 ng/L,平均值为144 ng/L)^[33]^,高于Ontario水中SCCPs的质量浓度(1.194 ng/L)^[34]^,后续需要重点关注该类化合物的海洋污染水平并开展长期监测。

**表4:** 渤海海域海水中有机氯农药、多氯联苯、短链氯化石蜡和德克隆的质量浓度

Sample	*α*-HCH	PCB 52	*anti*-DP	SCCPs
S1	0.2	-	-	69.7
S2	-	-	-	53.0
S3	-	-	-	130.6
S4	-	-	-	64.9
S5	-	-	-	76.0
S6	0.2	0.5	-	48.1
S7	-	-	-	43.0
S8	-	-	-	27.3
S9	-	-	-	35.1
S10	-	-	0.2	61.5
S11	-	-	-	27.5
S12	0.2	0.1	-	23.8
S13	0.2	0.1	-	50.5
S14	-	-	-	51.8
S15	0.3	-	-	40.0
S16	-	-	-	35.0
S17	-	-	0.3	-
S18	-	-	-	-
S19	0.3	0.7	-	19.5
S20	-	0.1	-	35.7

-: not detected.

## 3 结论

本研究以海水中4类高度受关注的含氯POPs为分析对象,建立了液液萃取-固相萃取前处理-GC-Orbitrap-HRMS分析方法。该方法前处理操作简便,灵敏度高,样品需求量少,可通过一次前处理实现海水中4类含氯POPs的分析,适用于大批量海水样本中多种含氯POPs的痕量检测,有望在海洋环境监测和应急监测海水污染领域发挥重要作用,为海洋生态文明建设和海洋可持续发展做出贡献。
